# Unraveling the Microbial Associations of Volatile Flavor Profiles in Spontaneously Fermented Camel Milk from Three Regions of Xinjiang, China

**DOI:** 10.3390/foods15162937

**Published:** 2026-08-21

**Authors:** Yating Wu, He Chen, Fulan Wang, Henigul Osman, Shiqi Zhang, Hongyan Zhang, Shuai Wang, Nan Zheng, Yankun Zhao

**Affiliations:** 1 Institute of Quality Standards & Testing Technology for Agro-Products, Xinjiang Academy of Agricultural Sciences, Urumqi 830091, China; wuyating0223@163.com (Y.W.); hechen@xaas.ac.cn (H.C.); fulanwang@xaas.ac.cn (F.W.); 13009693320@163.com (H.O.); zhangas_q@163.com (S.Z.); zhanghongyan08107@163.com (H.Z.);; 2 Key Laboratory of Functional Nutrition and Health of Characteristic Agricultural Products in Desert Oasis Ecological Region (Co-Construction by Ministry and Province), Ministry of Agriculture and Rural Affairs, Urumqi 830091, China; 3 Laboratory of Quality & Safety Risk Assessment for Agro-Products (Urumqi), Ministry of Agriculture and Rural Affairs, Urumqi 830091, China; 4 Xinjiang Key Laboratory of Agro-Products Quality & Safety, Urumqi 830091, China; 5 Key Laboratory for Quality & Safety Control for Milk and Dairy Products of Ministry of Agriculture and Rural Affairs, Institute of Animal Science, Chinese Academy of Agricultural Science, Beijing 100193, China

**Keywords:** spontaneously fermented camel milk, microbial diversity, volatile flavor compounds, correlation

## Abstract

Spontaneously fermented camel milk from Xinjiang is valued for its nutrition but faces quality inconsistency due to complex microbial ecosystems. This study investigated how regional microbial communities correlate with volatile flavor profiles. Nine samples from three distinct areas—Dabancheng, Hami, and Keping—were analyzed using bacterial 16S rRNA gene and fungal ITS region amplicon sequencing combined with comprehensive two-dimensional gas chromatography–time-of-flight mass spectrometry (GC×GC-TOF MS). Bacterial communities were dominated by *Firmicutes* and *Proteobacteria*, while *Ascomycota* and *Basidiomycota* prevailed among fungi. Dominant genera varied by region: *Lactobacillus* and *Dekkera* in Dabancheng; *Lactobacillus* and *Dipodascus* in Hami; *Lactococcus* and *Cutaneotrichosporon* in Keping. Flavor profiles also diverged, with Dabancheng being rich in esters, alcohols, and ketones; Hami featuring hydrocarbons and alcohols; and Keping containing elevated hydrocarbons, alcohols, and esters. Key statistical associations were identified: *Lactobacillus*, *Rahnella*, and *Acetobacter* were positively associated with propene and 3-decyn-2-ol (VIP > 1.0, |r| > 0.8), while *Pseudomonas*, *Lactococcus*, and *Klebsiella* showed negative correlations with vinyl n-caproate. These findings reveal statistical associations between geographic origin, specific microbial taxa, and flavor attributes, providing a scientific foundation that may inform future efforts toward developing targeted starter cultures pending microbial isolation and functional validation.

## 1. Introduction

Fermented camel milk, a unique naturally soured dairy product originating from Xinjiang, is highly regarded for its outstanding nutritional value and probiotic advantages. Nevertheless, its production remains largely artisanal, occurring in household settings. The spontaneous fermentation process presents substantial obstacles, including a complex microbial composition and inconsistent product quality, which significantly impede standardization and commercial advancement. Employing sequencing platforms like Illumina MiSeq, researchers have compared the microbial communities of naturally fermented camel milk from various locations—including Alxa and Sonid in Inner Mongolia as well as Xinjiang—highlighting the strong effect of geographic origin on microbial diversity [1]. A forward-looking direction in fermented food science is the combined use of metagenomics, metatranscriptomics, and metabolomics [2,3]. These multi-omics strategies have effectively clarified the associations between microbial community architecture, functional gene repertoires, and metabolic networks across different fermented foods [2], thus offering a useful methodological reference for investigations into fermented camel milk. For example, microbial metabolomics has been utilized to elucidate the biosynthetic routes of flavor compounds and to pinpoint the metabolic characteristics of microorganisms possessing a strong ability to generate aromas [4]. Furthermore, integrative omics approaches in food flavor research reveal that joint analyses merging microbiomics with metabolomics can efficiently determine the complete sensory profile of food items [5].

Regarding other fermented products, investigations have firmly demonstrated links between microbial ecology and the development of flavor. In the case of strong-flavor Baijiu, evidence shows that the succession of microbial populations within fermented grains (known as Jiupei) is intimately tied to flavor-related metabolism. Within this process, shifts in *Lactobacillus* populations constitute a complete metabolic pathway encompassing acid generation and ethanol production [6]. Likewise, during the fermentation of paocai, the association between patterns of microbial turnover and flavor chemicals is thoroughly recognized, wherein the metabolic functions of lactic acid bacteria are essential for generating distinctive taste attributes [7]. In fermented soybean paste, or doujiang, the progression of microbes and their metabolic activities across different fermentation stages dictate the ultimate sensory characteristics. Here, the varieties and concentrations of amino acids such as aspartic acid and glutamic acid are tightly connected to microbial diversity [8]. Approaches to studying the microbial ecology of traditional Chinese fermented foods highlight the application of metagenomics to uncover flavor-associated metabolic functional genes and to forge links between particular microorganisms and critical metabolic products [9].

In contrast, research on fermented camel milk, especially studies that systematically elucidate how geographically distinct microbial communities mechanistically drive the formation of specific volatile flavor profiles, lags behind that conducted on other more extensively studied fermented foods, especially with respect to the combined application of metagenomic and metabolomic approaches. Existing research remains mostly confined to the preliminary phase of characterizing fundamental microbial communities. The comprehensive mechanistic connections linking geographical location, microbial assemblages, and distinctive flavor profiles have yet to be clarified. Consequently, there is an absence of a solid theoretical foundation in microbial ecology to support targeted flavor modification and standardized manufacturing. Accordingly, the present investigation intends to apply integrated microbiome analysis and volatile compound profiling analyses to thoroughly decode the microbial ecosystem configuration of fermented camel milk sourced from various areas within Xinjiang and to clarify how it relates to characteristic flavor metabolic pathways. This effort aims to fill the existing void in knowledge surrounding microbial ecology and flavor biochemistry during the natural fermentation of camel milk, while simultaneously supplying essential data to facilitate the creation of starter cultures and the standardization processes. The outcomes are anticipated to carry theoretical and practical significance for transitioning the camel milk sector away from a conventional, experience-driven approach and toward a precision fermentation model steered by microbiota insights.

## 2. Materials and Methods

### 2.1. Sample Collection

A total of nine specimens of naturally fermented camel milk were gathered from three different areas within Xinjiang, China: Dabancheng (designated as A), Hami (designated as B), and Keping (designated as C). Each sample was obtained from a different household producer within the same region. For each region, three separate samples were obtained (see Table 1). All samples were produced through traditional spontaneous fermentation. Every production site, each being a farm from which a sample originated, kept a herd consisting of no fewer than 50 Bactrian camels. From each site, 500 mL of milk was collected, representing a single production batch from that particular farm. Immediately after collection, all specimens were placed into sterile sampling containers, shipped on dry ice, and upon reaching the laboratory, kept at −80 °C until subsequent analysis was performed.

### 2.2. Reagents and Instruments

Analytical-grade ethanol was procured from Aladdin Biochemical Technology Co., Ltd., located in Shanghai, China. The deuterated internal standard known as n-hexyl-d13 alcohol was sourced from C/D/N Isotopes Inc., based in Pointe-Claire, QC, Canada. A solid-phase microextraction (SPME) fiber assembly, featuring a 50/30 μm coating of divinylbenzene/carboxen/polydimethylsiloxane (DVB/CAR/PDMS) and a length of 1 cm, was acquired from Supelco in Bellefonte, PA, USA. Ultrapure water (H_2_O) was generated using a Milli-Q water purification system manufactured by Millipore in Bedford, MA, USA. The analysis of volatile compounds was conducted on a two-dimensional gas chromatograph (Agilent 8890A GC; Agilent Technologies, Palo Alto, CA, USA) that was interfaced with a time-of-flight mass spectrometer (LECO Pegasus BT 4D TOF-MS; LECO, St. Joseph, MI, USA).

### 2.3. Sequencing Methods and Data Analysis

Amplification was carried out on the bacterial 16S rRNA gene and the fungal internal transcribed spacer (ITS) gene regions, after which high-throughput sequencing was performed using the Illumina HiSeq 2500 sequencing platform, Illumina, Inc., San Diego, CA, USA. Total genomic DNA was extracted from 200 mg of each sample using the PowerSoil Pro DNA Isolation Kit (Qiagen, Germany) following the manufacturer’s protocol. A negative control (sterile water) was included in each extraction batch. The bacterial 16S rRNA gene V3–V4 hypervariable region was amplified using primers 338F (5′-ACTCCTACGGGAGGCAGCAG-3′) and 806R (5′-GGACTACHVGGGTWTCTAAT-3′). The fungal ITS1 region was amplified using primers ITS1F (5′-CTTGGTCATTTAGAGGAAGTAA-3′) and ITS2R (5′-GCTGCGTTCTTCATCGATGC-3′). PCR amplification was performed in triplicate 25 μL reactions containing 12.5 μL 2 × Taq Plus Master Mix, 1 μL each primer (10 μM), 1 μL template DNA, and 9.5 μL nuclease-free water. Thermal cycling conditions were: initial denaturation at 94 °C for 3 min; 35 cycles of 94 °C for 30 s, 55 °C (16S) or 52 °C (ITS) for 30 s, and 72 °C for 45 s; final extension at 72 °C for 10 min. Negative controls (no-template) were included in all PCR runs. Initially, the raw paired-end reads derived from sequencing were combined according to their overlapping segments with FLASH (version 1.2.11). Following this, quality control and filtration of the sequences were executed using Trimmomatic (version 0.33). Subsequently, chimeric sequences were identified and eliminated through UCHIME (version 8.1), resulting in high-quality, usable sequences. These refined sequences were then grouped into operational taxonomic units (OTUs) at a 97% similarity cutoff with USEARCH (version 10.0). Taxonomic assignment of the OTUs was achieved by comparing them against the SILVA database (release 132), applying a confidence threshold of 0.7. Taxonomic assignment of bacterial ASVs was performed against the SILVA 138.1 database. Fungal ASVs were classified using the UNITE 9.0 database (dynamic release, 31 January 2024). Drawing from the OTU clustering outcomes, sequencing depth (via rarefaction) and microbial alpha-diversity metrics, including Chao1 and Shannon indices, were determined for every specimen. Amplicon libraries were prepared using the NEBNext Ultra II DNA Library Prep Kit (New England Biolabs, Ipswich, MA, USA). Paired-end sequencing (2 × 250 bp for 16S; 2 × 300 bp for ITS) was performed on an Illumina NovaSeq 6000 platform (Illumina, USA). Sequencing depth averaged 62,348 ± 8215 reads per sample for 16S and 58,176 ± 9432 reads per sample for ITS. Quality filtering was applied with a maximum expected error threshold of 1.0 and a minimum read length of 200 bp. All bioinformatic processing was carried out within the QIIME2 framework (version 2023.5). Raw sequencing reads were processed using a combination of tools integrated within the QIIME2 2024.2 framework. Specifically, FLASH v1.2.11 was used to merge paired-end reads, Trimmomatic v0.39 was used for quality trimming (sliding window of 4 bp with average quality threshold Q20), and UCHIME v2.4 was used for chimera detection and removal via the USEARCH v11.0 algorithm. Denoising was performed using DADA2 within QIIME2 to generate amplicon sequence variants (ASVs). All downstream analyses (alpha/beta diversity, taxonomic composition) were conducted within the QIIME2 environment.

### 2.4. Comprehensive Two-Dimensional Gas Chromatography–Time-of-Flight Mass Spectrometry (GC×GC-TOFMS)

#### 2.4.1. GC×GC Analysis

Volatile compounds were extracted using headspace solid-phase microextraction (HS-SPME). Five milliliters of each fermented camel milk sample were placed in a 20 mL glass vial containing 1.5 g NaCl and 10 μL of 2-methyl-3-heptanone (0.816 mg/mL in methanol) as internal standard. The vial was sealed with a PTFE-silicone septum cap and equilibrated at 60 °C for 15 min with continuous agitation (500 rpm). A 50/30 μm divinylbenzene/carboxen/polydimethylsiloxane (DVB/CAR/PDMS) SPME fiber (Supelco, Bellefonte, PA, USA) was exposed to the headspace for 40 min at 60 °C with agitation. After extraction, the fiber was immediately desorbed in the GC injector port at 260 °C for 5 min in splitless mode. Measurements were carried out on a LECO Pegasus^®^ 4D instrument (LECO, St. Joseph, MI, USA). This setup included an Agilent 8890A gas chromatograph (Agilent Technologies, Palo Alto, CA, USA) fitted with a split/splitless injector and a dual-stage cryogenic modulator (LECO), all linked to a time-of-flight mass spectrometry (TOFMS) detector (LECO). For the first dimension (^1^D), a DB-HeavyWax capillary column was employed, measuring 30 m in length with a 250 µm internal diameter and a 0.5 µm film thickness (Agilent, Santa Clara, CA, USA). The second-dimension (^2^D) column consisted of an Rxi-5Sil MS capillary column, 2.0 m long with a 150 µm internal diameter and a 0.15 µm film thickness (Restek, Bellefonte, PA, USA). Ultra-high-purity helium (purity > 99.999%) served as the carrier gas, delivered at a steady flow rate of 1.0 mL/min.

The temperature programming for the GC oven proceeded as follows: an initial hold at 40 °C lasting 3 min, followed by a ramp to 150 °C at 4 °C per minute with a 2 min hold, then an increase to 200 °C at 6 °C per minute, and finally a rise to 230 °C at 10 °C per minute, concluding with a 10 min hold. The secondary oven was maintained at a temperature 5 °C higher than that of the primary oven. Meanwhile, the modulator was kept 15 °C above the secondary oven temperature. A modulation cycle of 5.0 s was applied throughout. The injector temperature of the GC was set to 250 °C [10,11].

#### 2.4.2. Mass Spectrum Conditions

The analysis of volatile compounds was conducted on the LECO Pegasus BT 4D time-of-flight mass spectrometer. Both the transfer line and the ion source were held at a temperature of 250 °C. Spectral data were collected at a rate of 200 scans per second. The mass spectrometer functioned in electron ionization (EI) mode, applying an ionization energy of 70 eV. The mass-to-charge ratio (*m*/*z*) scanning window was configured from 35 to 550, and the detector voltage was set to 1984 V [10,11].

### 2.5. Data Analysis

Information pertaining to the samples, such as compound identities and their corresponding CAS registry numbers, was supplied by the databases. These pieces of data were combined to derive the conclusive analytical outcomes. In order to facilitate comparisons among datasets with differing scales, the raw measurements were standardized through an internal standard technique. Compound identification was performed by comparing mass spectra with the NIST 2020 library. Identification criteria were: (i) spectral match factor ≥ 800 (out of 999), (ii) retention index (RI) deviation ≤ 20 units from published RI values on a DB-WAX column, and (iii) visual inspection of chromatographic peak shape. When available, authentic standards were used for confirmation. Only compounds detected in all three replicates of at least one sample group were retained for statistical analysis. The subsequent processing of the data involved hierarchical cluster analysis (HCA); multivariate statistical methods, including principal component analysis (PCA); and flavoromics evaluations, such as correlation network analysis examining the relationships between volatile constituents and microbial populations.

All statistical analyses were performed using R software (v4.3.2, R Foundation for Statistical Computing, Vienna, Austria). Alpha-diversity indices (Shannon, Simpson, Chao1, Pielou evenness) were calculated using the vegan package (v2.6-4). Differences in alpha diversity among regions were assessed using one-way ANOVA followed by Tukey’s HSD post hoc test when normality assumptions were met; otherwise, the Kruskal–Wallis test was used. Beta-diversity was visualized using principal coordinate analysis (PCoA) based on Bray–Curtis dissimilarity, and permutational multivariate analysis of variance (PERMANOVA, adonis2, 999 permutations) was used to test for significant differences among regions. Differential abundance analysis of microbial taxa and volatile compounds among regions was performed using linear discriminant analysis effect size (LEfSe, LDA score threshold > 2.0, *p* < 0.05) and one-way ANOVA with false discovery rate (FDR) correction (Benjamini–Hochberg method). Correlation analysis between dominant microbial genera (relative abundance > 1% in at least one sample) and volatile compounds was performed using Spearman’s rank correlation coefficient, implemented in the psych package (v2.3.9). *p*-Values were adjusted using the Benjamini–Hochberg FDR correction, and only correlations with r > 0.7 and adjusted *p* < 0.05 were considered significant. Partial least squares regression (PLSR) was performed using the pls package (v2.8-3) with microbial genera as X-variables and volatile compounds as Y-variables. Variable importance in projection (VIP) scores were calculated, and variables with VIP > 1.0 were considered important contributors. Model performance was evaluated using leave-one-out cross-validation (LOOCV), and the root mean square error of cross-validation (RMSECV) and cumulative explained variance (R^2^X and R^2^Y) were reported.

## 3. Results

### 3.1. Analysis of Differences in the Composition of Bacterial Microbial Communities

#### 3.1.1. Analysis of Microbial Diversity Differences

**Figure 1:**
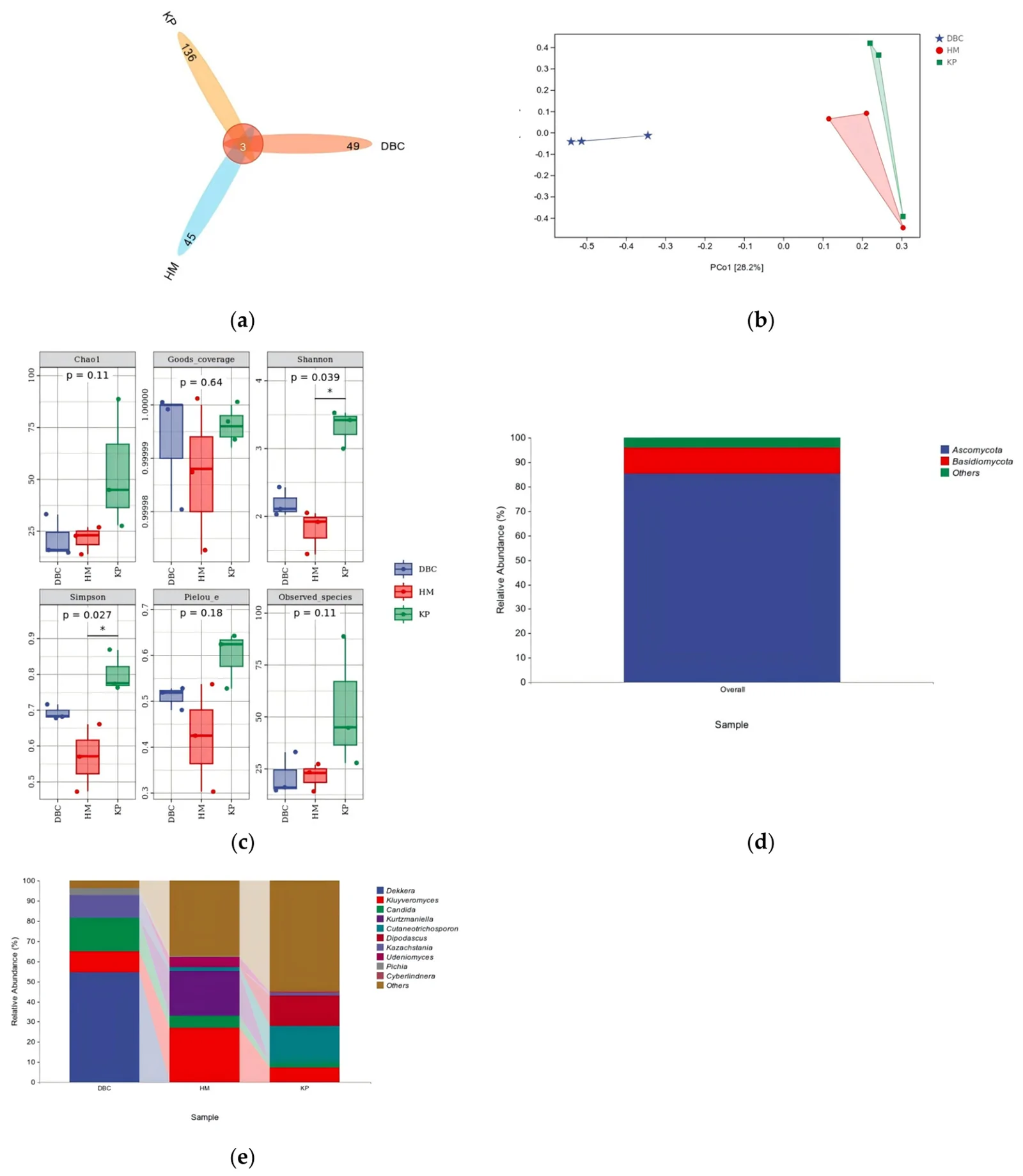
Differential analysis of bacterial microbial communities and diversity. (**a**) Venn diagram analysis among groups; (**b**) PCA analysis; (**c**) box plots of alpha diversity; the relative abundance of bacteria at the phylum level (**d**) and genus level (**e**). * indicates statistical significance (*p* < 0.05) based on pairwise comparisons.

To explore how microbial composition varies by location, the bacterial communities present in fermented camel milk specimens collected from three different areas were examined using high-throughput sequencing. In Figure 1a, a Venn diagram illustrates the count of operational taxonomic units (OTUs) that are either shared or unique across the three sample sets. Across all specimens, a total of 1114 OTUs were detected. Of these, merely 3 OTUs were found in every region, whereas 337 OTUs were exclusive to the Dabancheng group, 391 to the Hami group, and 383 to the Keping group. This finding suggests that the bacterial community in the Keping samples possesses greater richness compared to the other two locations.

Evaluation of alpha diversity (Figure 1c) indicated that although the Chao1 and Faith’s PD indices did not differ significantly among the three regions (*p* > 0.05), clear numerical trends were apparent. Based on the Chao1 index, bacterial richness did not differ significantly among the three regions (*p* > 0.05, one-way ANOVA). However, the Venn diagram revealed that Hami harbored the highest number of unique bacterial ASVs (187), followed by Keping (156) and Dabancheng (132), suggesting region-specific taxa despite comparable overall richness. The Keping samples consistently exhibited the highest mean values for both the Chao1 (richness) and Shannon (diversity) indices, surpassing those from Dabancheng and Hami. In contrast, the Hami samples exhibited the lowest mean values for these indices. All samples attained 100% sequencing coverage, confirming that the depth of sequencing was adequate to encompass the extant bacterial diversity; consequently, the results faithfully represent the native bacterial populations. The Pielou evenness metric revealed that bacterial community evenness was notably higher in specimens from Keping and Dabancheng relative to those from Hami (*p* = 0.018). On the whole, the bacterial alpha diversity values for the Hami samples tended to be lower than those observed for the Keping and Dabancheng groups.

#### 3.1.2. Analysis of Bacterial Community Composition in Fermented Camel Milk

To evaluate how similar the microbial communities were across different specimens, beta diversity analysis was carried out. Following this, principal coordinate analysis (PCoA) was applied to examine variations in bacterial makeup among the three locations. The resulting PCoA plot (displayed in Figure 1b) reveals a distinct separation according to the region of origin, with principal coordinate 1 (PCoA1) accounting for 63.7% of the total variance and principal coordinate 2 (PCoA2) explaining 20.5%. As illustrated in the figure, the bacterial populations from Keping samples formed a tight cluster, pointing to a high degree of similarity among specimens from that area. On the other hand, samples originating from Hami and Dabancheng exhibited a more scattered and separated arrangement, implying greater variability in bacterial community structure among individual samples within those two regions.

The prevailing bacterial phyla across every specimen are presented in Figure 1d. The microbial communities were predominantly made up of *Firmicutes* and *Proteobacteria*. When examined at the genus level (Figure 1e), the makeup and organization of bacterial populations showed marked differences among the areas. In the Dabancheng specimens, the genera with the highest abundances were *Lactobacillus* (60.44%), *Lactococcus* (14.66%), and *Rahnella* (12.06%). For the Hami samples, the leading genera consisted of *Lactobacillus* (32.27%), *Lactococcus* (28.86%), and *Pseudomonas* (12.56%). In the Keping samples, *Lactococcus* (43.80%) and *Serratia* (16.01%) were the most prevalent. These observations indicate that, although certain bacterial genera, such as *Lactobacillus* and *Lactococcus*, are shared across all regions, their proportional abundances along with the occurrence of other dominant taxa display clear location-specific trends.

### 3.2. Analysis of Differences in Fungal Community Composition

#### 3.2.1. Analysis of Fungal Diversity

**Figure 2:**
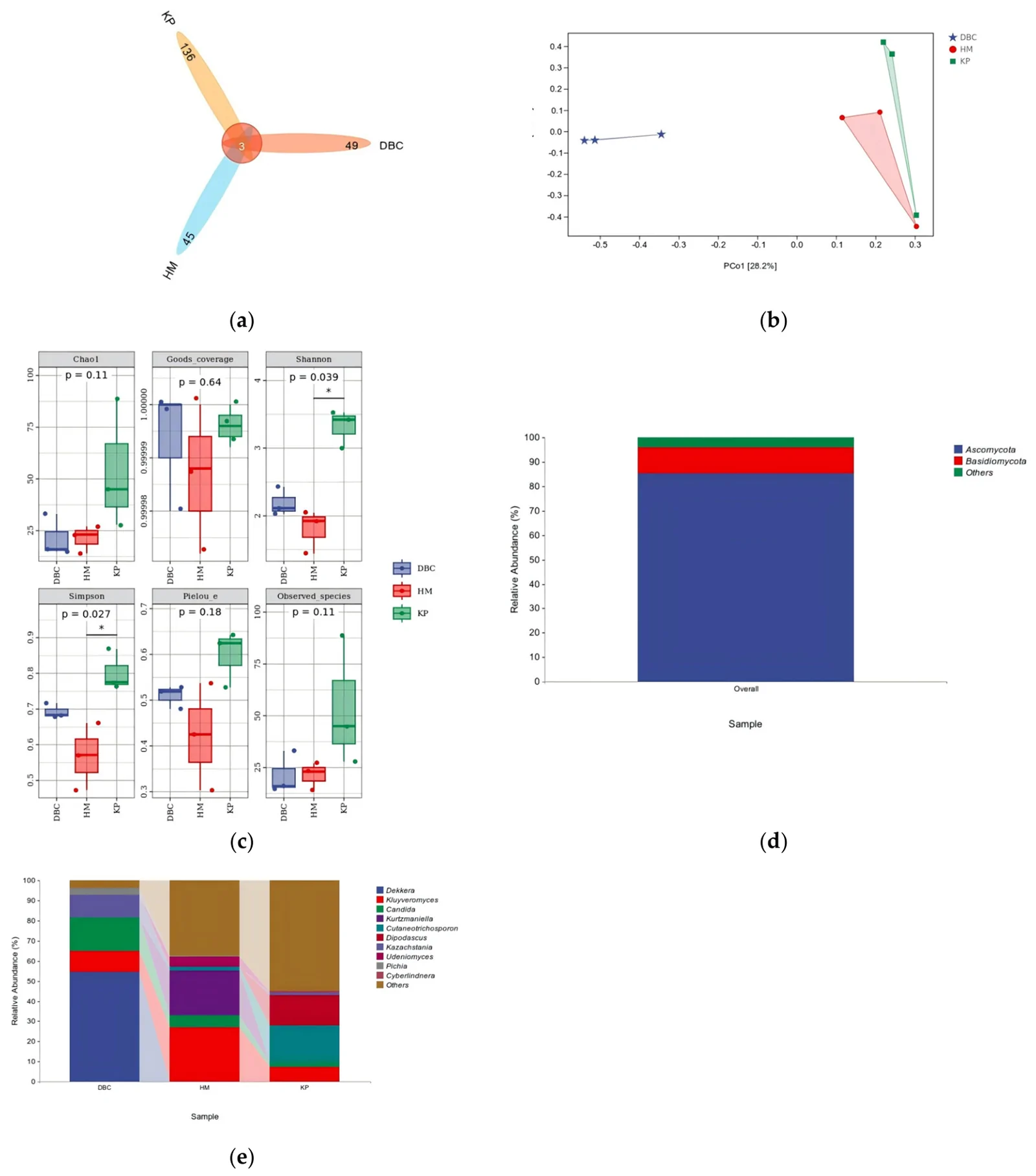
Differential analysis of fungal microbial communities and diversity. (**a**) Venn diagram analysis among groups; (**b**) PCA analysis; (**c**) box plots of alpha diversity; the relative abundance of fungi at the phylum level (**d**) and genus level (**e**). * indicates statistically significant differences (*p* < 0.05) based on Duncan’s multiple range test.

Variations in the composition of fungal communities across samples from different areas were examined using high-throughput sequencing targeting the ITS gene region. A Venn diagram displaying the count of operational taxonomic units (OTUs) for each group is provided in Figure 2a. In total, 233 OTUs were detected across all specimens. Out of these, three OTUs were common to every region, whereas 49 OTUs were exclusive to Dabancheng, 45 to Hami, and 136 to Keping. This outcome points to a greater level of fungal richness in the Keping specimens relative to the other two locations.

Assessment of alpha diversity metrics (shown in Figure 2c) revealed that all samples achieved 100% sequencing coverage, verifying that the sequencing depth was adequate to encompass the existing fungal diversity. The Keping samples exhibited the greatest fungal richness and diversity, surpassing the values recorded for the Dabancheng and Hami groups. Additionally, the Pielou evenness metric demonstrated that fungal community evenness was considerably higher in specimens from Keping and Dabancheng compared to those from Hami (SD = 0.018). Taken together, the fungal alpha diversity values for the Hami samples were typically lower than those observed for the Keping and Dabancheng groups.

#### 3.2.2. Analysis of Fungal Community Composition in Fermented Camel Milk

To compare variations in fungal community composition across the different areas, principal component analysis (PCA) was carried out. The resulting PCA plot (presented in Figure 2b) demonstrates segregation according to the sampling location, with principal coordinate 1 (PCoA1) capturing 28.2% of the total variance, and principal coordinate 2 (PCoA2) accounting for 18.7%. As depicted, the fungal populations from Dabancheng specimens formed a compact cluster, reflecting a high degree of resemblance among samples originating from that area. Conversely, samples collected from Hami and Keping showed a more scattered and separate arrangement, indicating greater variability in fungal community structure across individual specimens within these two locations.

When examined at the phylum level, the fungal communities in every sample were chiefly constituted by *Ascomycota* and *Basidiomycota* (Figure 2d). Marked disparities in makeup and organization became evident at the genus level (Figure 2e). Within the Dabancheng samples, the genera with the highest abundances were *Dekkera* (54.49%), *Candida* (16.45%), and *Kazachstania* (11.35%). For the Hami samples, the predominant genera were *Kazachstania* (27.12%) and *Kurtzmaniella* (22.31%). In the Keping samples, *Cutaneotrichosporon* (18.06%) and *Dipodascus* (14.96%) were the most prevalent. These observations suggest that, although all fungal communities originate from the same leading phyla, the particular dominant fungal genera and their proportional abundances display unmistakable location-dependent patterns.

### 3.3. Analysis of Differences in Flavor Compound Composition

#### 3.3.1. Statistics of Identified Flavor Compounds

**Figure 3:**
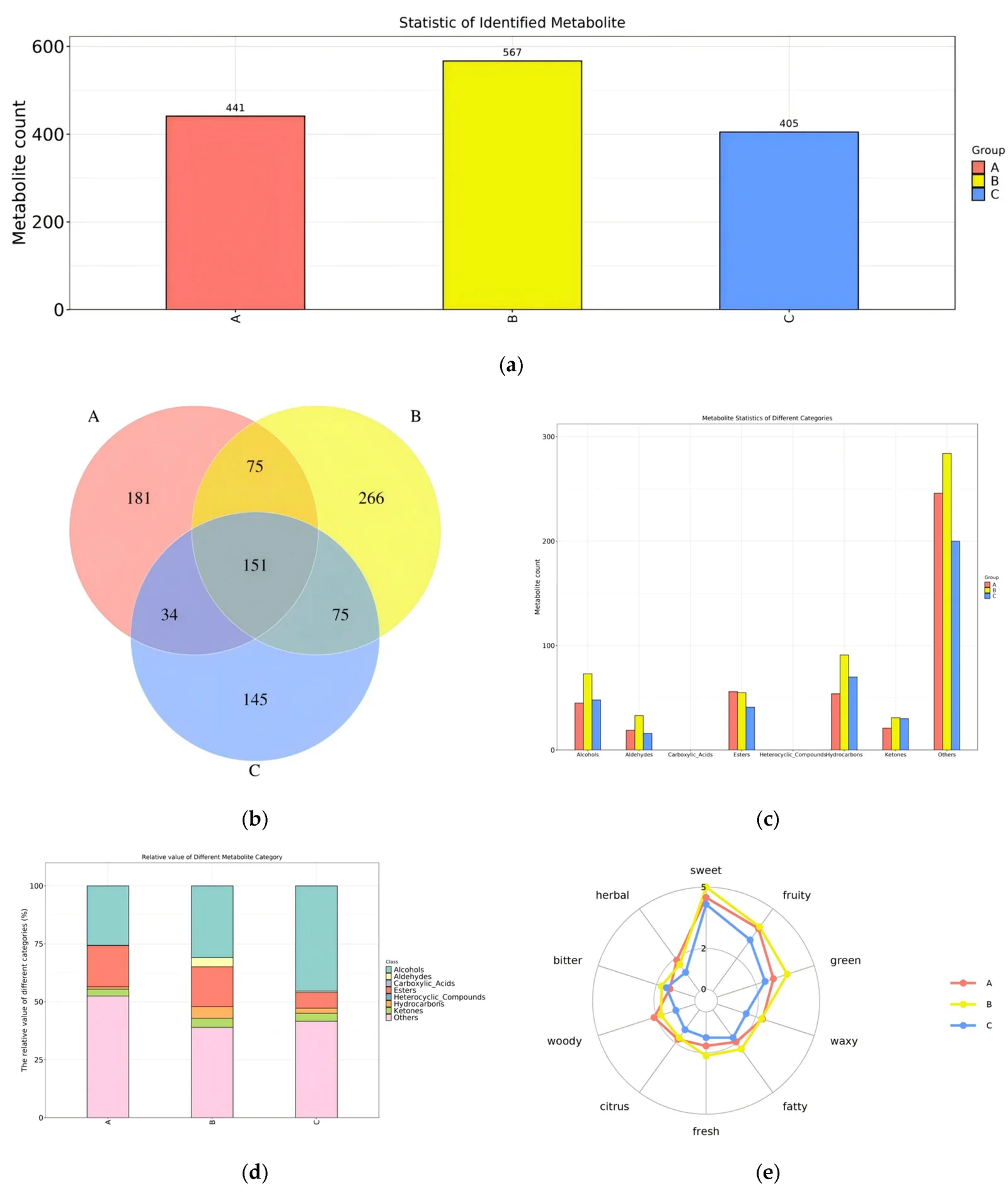
(**a**) Statistic of identified compounds; (**b**) UpSetPlot of identified compounds; (**c**) metabolite statistics of different categories; (**d**) stacked bar chart of the relative values of different compound classes; (**e**) sensory flavor profile radar map.

The unprocessed data for volatile flavor compounds were handled with ChromaTOF software (Version 5.0), where compound identification relied on spectral comparisons against the NIST 2020 library. Following the removal of background noise, a catalog of identified flavor constituents was assembled. The total number of compounds detected in samples from each location is shown in Figure 3a. Samples from Hami yielded the highest number of identified flavor compounds, followed by Dabancheng, while Keping samples had the fewest.

The allocation of exclusive and common flavor compounds among the sample categories was depicted through an UpSet plot (Figure 3b). Hami specimens possessed the largest number of compounds found only in that group (266), whereas Keping samples had the fewest unique compounds (145). A total of 151 flavor compounds were found to be present in specimens from every region.

#### 3.3.2. Classification of Flavor Compounds

Figure 3c illustrates the quantity of flavor compounds categorized under each primary chemical class. Across all specimens, the principal categories identified encompassed alcohols, aldehydes, carboxylic acids, esters, heterocyclic compounds, hydrocarbons, and ketones. When comparing the different areas, Dabancheng samples exhibited the greatest counts of esters, hydrocarbons, alcohols, and ketones. Hami specimens contained the largest numbers of hydrocarbons and alcohols, whereas Keping samples showed the highest quantities of hydrocarbons, alcohols, and esters.

The proportional amounts of these flavor compound classes differed across the locations, as depicted in Figure 3d. Alcohols were present at comparatively high levels in specimens from every region. Esters were demonstrated to have a greater relative abundance in Dabancheng and Hami samples compared to those from Keping. Carboxylic acids, including acetic acid, nonanoic acid, and hexanoic acid, were detected, albeit at low relative abundances (<2% of total peak area). Neither carboxylic nor heterocyclic compounds were identified in any of the specimens collected from the three areas.

#### 3.3.3. Odor Activity Analysis of Volatile Compounds

The odor activity value (OAV) was calculated as the ratio of the concentration of each volatile compound to its respective odor detection threshold in water reported in the literature. Compounds with OAV ≥ 1 were considered to contribute to the overall aroma profile. In situations where specimens contain a large variety of flavor constituents, the relative percentage composition is employed as a substitute for absolute concentration measurements. Under these circumstances, the relative odor activity value (ROAV) is utilized to assess the contribution made by each volatile compound to the complete aromatic character. The ROAV is determined using the following formula:

(1) $${ROAV}_{B}=100\times \frac{{P\mathrm{eak}}_{B}}{T_{B}}/\frac{{Peak}_{A}}{T_{A}}$$

In this formula, TA and PeakA refer, respectively, to the odor threshold and the chromatographic peak area of the substance possessing the lowest odor threshold within the specimen (this compound is assigned a baseline ROAV of 100). Meanwhile, TB and PeakB denote the odor threshold and peak area, respectively, of the substance under assessment. Any compound is regarded as a significant aroma contributor whenever its computed ROAV equals or exceeds 1.

According to the data presented in Table 2, Hami specimens contained the greatest quantity of key aroma compounds, nine in total, while both Dabancheng and Keping samples had the smallest number, with only four each. When considering every specimen collectively, the substances attaining the highest ROAV values were 2,3-butanedione (buttery aroma), 2-pentylfuran (fruity, green, earthy notes), and (E)-2-nonenal (fatty, cucumber-like, waxy character). It is noteworthy that 2,3-butanedione consistently registered the maximum ROAV across all sample groups examined, underscoring its dominant role in imparting a buttery sensory note. Among alcohols, 1-octen-3-ol contributed a mushroom-like, earthy aroma, while 3-methyl-1-butanol imparted a malty, whiskey-like note. Key esters, such as ethyl heptanoate and ethyl nonanoate, were associated with fruity, pineapple-like aromas and floral, rose-like aromas, respectively, highlighting their contribution to the sweet and fruity flavor profile observed in Dabancheng samples.

#### 3.3.4. Flavor Characteristic Analysis

The flavor of milk encompasses both well-defined taste and odor qualities as well as intricate, indivisible sensory characteristics. To characterize the sensory properties of the volatile compounds present in the fermented camel milk specimens, sensory descriptors for volatile compounds were obtained from the FlavorDB database and peer-reviewed literature. These database-derived descriptors should not be interpreted as experimentally measured sensory characteristics of the samples themselves, but rather as a repository comprising 25,595 flavor molecules linked to an extensive array of tastes and scents. For each specimen, the flavor was characterized using ten sensory descriptors: sweet, fruity, green, waxy, fatty, woody, fresh, citrus, herbal, and floral. Radar charts illustrating the flavor profiles for each sample category are provided in Figure 3e. The findings indicate that the majority of specimens exhibit comparable flavor patterns, with the most notable distinctions appearing in the herbal and woody categories. The predominant flavor characteristics observed across the fermented camel milk samples were sweet, fruity, and green.

#### 3.3.5. Multivariate Statistical Analysis

**Figure 4:**
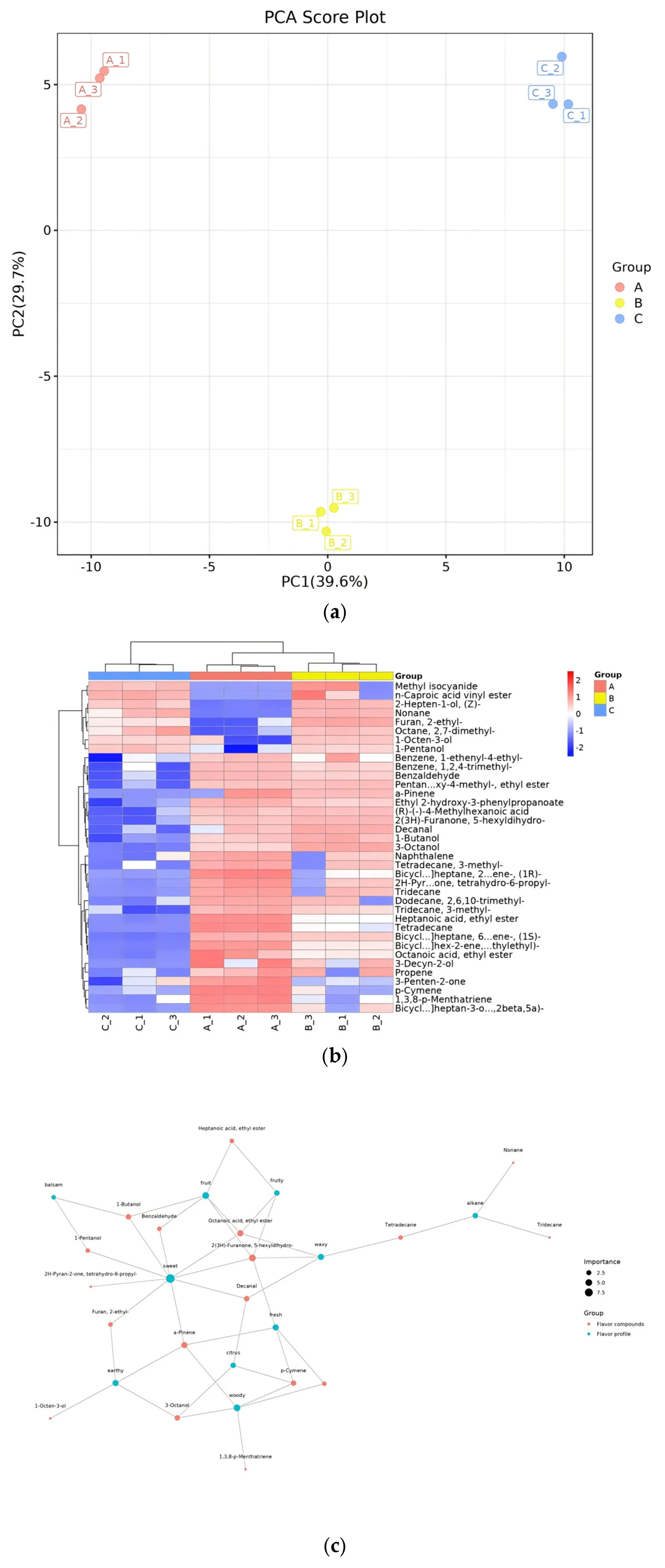
(**a**) PCA score plot; (**b**) hierarchical clustering heatmap of differential compounds; (**c**) the network of sensory flavor characteristics and flavor compounds.

Principal component analysis (PCA) was utilized as a multivariate statistical technique to evaluate the variability among specimens, thereby streamlining the data framework and illuminating relationships between samples. In this investigation, the initial two principal components, PC1 and PC2, collectively accounted for 69.3% of the overall variance. The outcomes of the PCA are depicted in Figure 4a. The specimens were segregated into two clear groupings: samples from Keping formed a distinct cluster, whereas those from Dabancheng and Hami grouped together.

#### 3.3.6. Analysis of Differential Compounds

**Figure 5:**
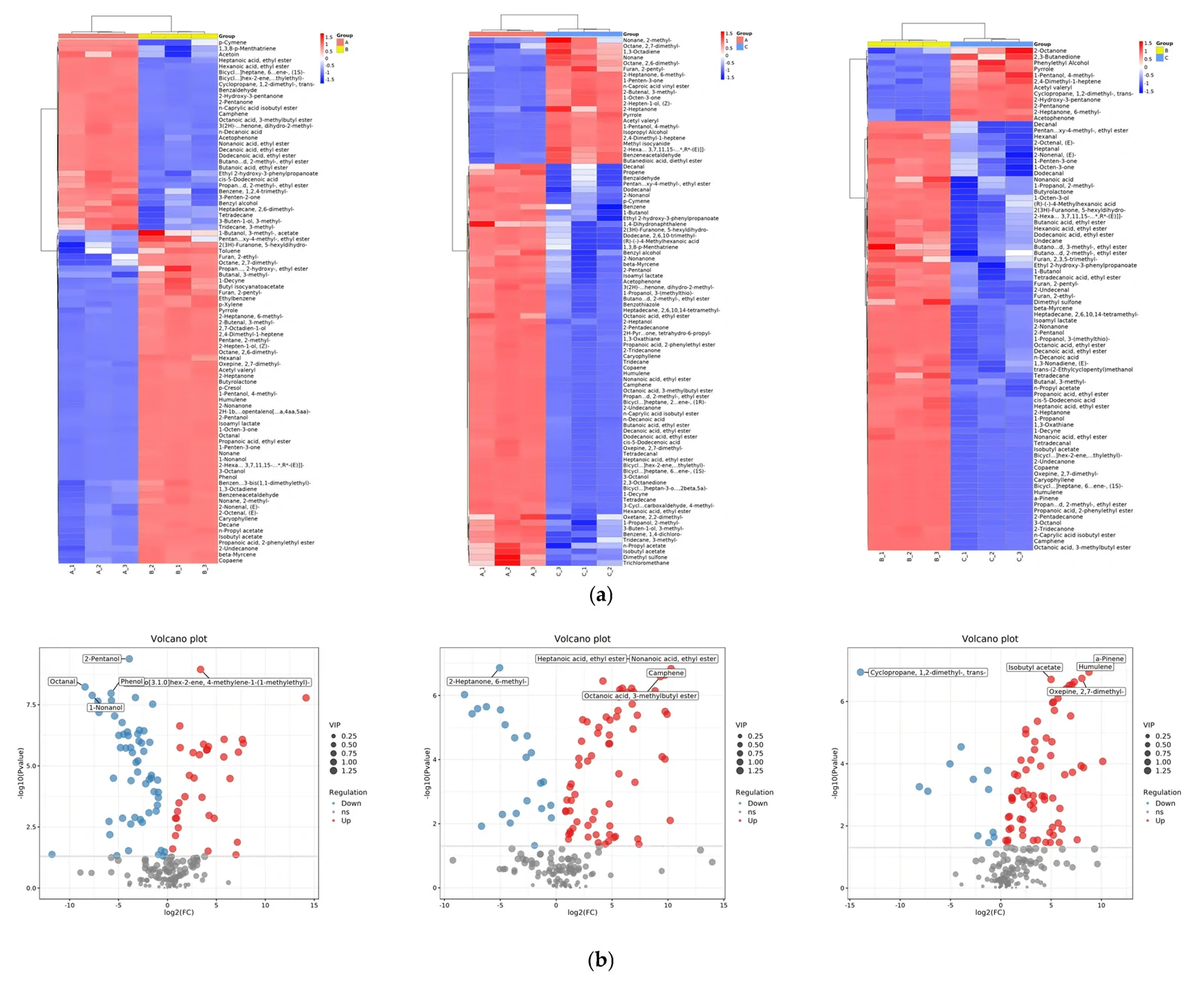
(**a**) Heatmap of the hierarchical clustering of different regions; (**b**) dynamic multi-group scatter plots of different compounds.

Differences in volatile compound relative abundances among the three regions were assessed using one-way ANOVA with Tukey’s HSD post hoc test. *p*-Values were adjusted using the Benjamini–Hochberg FDR correction, and adjusted *p* < 0.05 was considered statistically significant, along with a variable importance in projection (VIP) score exceeding 1. In total, 37 such compounds were recognized, and their proportional abundances are illustrated in a heatmap (Figure 4b), which emphasizes the disparities among the different sample categories. The heatmap reveals that specimens from Dabancheng and Hami contained notably greater levels of several of these substances relative to those from Keping. This finding is consistent with the PCA outcomes, indicating that these compounds serve as major determinants of the flavor distinctions observed across locations. Furthermore, side-by-side contrasts were carried out to detect substances whose abundances varied specifically between two given regions; the outcomes of these analyses are displayed in dedicated heatmaps (for instance, Figure 5a).

For deeper examination, differential substances were filtered using a dynamic multi-group scatter plot, applying thresholds of a fold change (FC) greater than 1.5 or less than 0.67. This graphical representation (Figure 5b) illustrates the patterns of metabolite up-regulation and down-regulation observed in pairwise comparisons between regions. The count of differential flavor compounds amounted to 88 (with 32 increased and 56 decreased) for the Dabancheng versus Hami comparison, 92 (70 increased and 22 decreased) for Dabancheng versus Keping, and 82 (70 increased and 12 decreased) for Hami versus Keping. The scatter plot reveals that the majority of differential substances were present at higher levels in the first region listed in each comparison pair.

Certain compounds displayed particularly notable expression trends. Specifically, 2-pentanol occurred at markedly lower concentrations in Dabancheng specimens relative to those from Hami. 6-Methyl-2-heptanone was substantially reduced in Dabancheng compared to Keping. Trans-1,2-dimethylcyclopropane was considerably less abundant in Hami than in Keping. On the other hand, 4-methylene-1-(1-methylethyl) bicyclo [3.1.0] hex-2-ene and trans-1,2-dimethylcyclopropane were present at significantly elevated levels in Dabancheng relative to Hami. Ethyl heptanoate and ethyl nonanoate were found in much greater quantities in Dabancheng than in Keping, whereas α-pinene and humulene were considerably more abundant in Hami than in Keping.

#### 3.3.7. Flavor Characterization of Differential Compounds

A network diagram depicting the connections between flavor substances and their associated sensory properties was created using Igraph in conjunction with FlavorDB. This visualization (Figure 4c) showcases the 10 most commonly occurring flavor descriptors along with the 18 volatile compounds that are associated with them. By applying the criterion of a relative odor activity value (ROAV) equal to or greater than 1, the principal aroma compounds that showed differential abundance were determined for each location. For the Dabancheng specimens, the key substances comprised 2,3-butanedione, α-pinene, 2-pentylfuran, and (E)-2-nonenal. In the Hami samples, nine significant compounds were recognized: (E)-2-nonenal, 2,3-butanedione, heptanal, 2-pentylfuran, (E)-2-octenal, 1-octen-3-one, acetic acid, α-pinene, and 2-undecanone. The Keping samples contained four essential compounds: 2,3-butanedione, 2-pentylfuran, 1-octen-3-one, and (E)-2-nonenal.

### 3.4. Correlation Analysis Between Microbial Communities and Flavor Compounds

**Figure 6:**
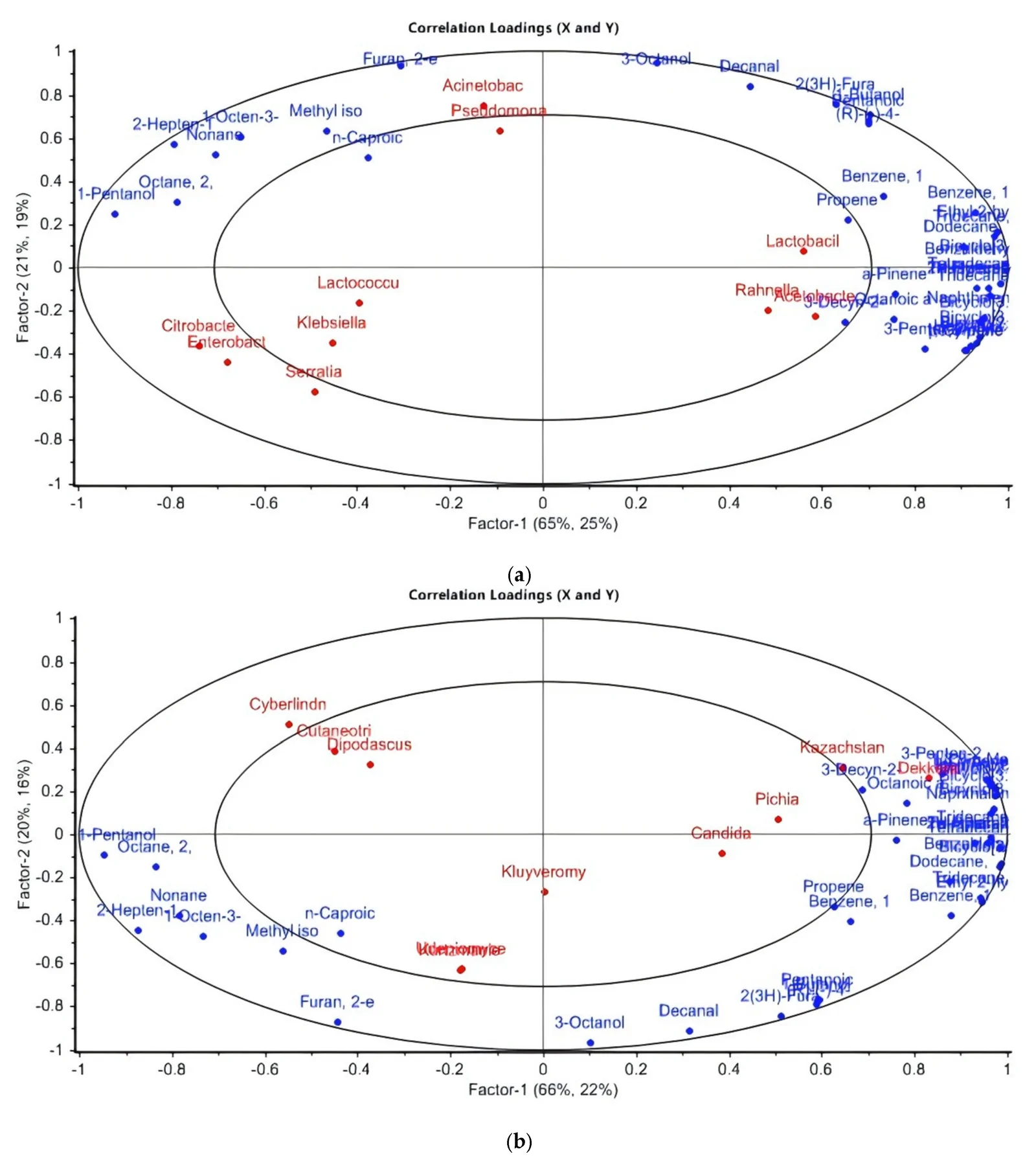
(**a**) Correlation analysis between bacteria and flavor substances; (**b**) correlation analysis between fungi and flavor substances.

To investigate the role of bacteria in flavor formation, partial least squares regression (PLSR) analysis was conducted on bacterial communities and differential flavor compounds in fermented camel milk from different regions. A regression model was established with the relative abundance of the top 10 bacterial genera as the independent variable and the content of flavor compounds as the dependent variable to analyze their correlations. In the resulting PLSR plots (Figure 6), the inner and outer ellipses represent 50% and 100% of the explained variance, respectively. Variables positioned between these ellipses indicate that the model provides a good explanatory power for the relationship.

Figure 6a shows that vinyl n-caproate was significantly negatively correlated with *Pseudomonas*, *Lactococcus*, and *Klebsiella*. In contrast, propene and 3-decyn-2-ol were significantly positively correlated with *Lactobacillus*, *Rahnella*, and *Acetobacter*. This suggests that *Lactobacillus*, *Rahnella*, and *Acetobacter* are the principal bacterial genera contributing to the differential flavor formation in fermented camel milk across regions. *Acinetobacter*, *Citrobacter*, *Enterobacter*, and *Serratia* showed no strong correlation with the differential flavor compounds.

Similarly, Figure 6b indicates that vinyl n-caproate was significantly negatively correlated with the fungal genera *Cutaneotrichosporon*, *Dipodascus*, and *Udeniomyces*. Propene and 3-decyn-2-ol were significantly positively correlated with *Kluyveromyces*, *Candida*, *Kazachstania*, and *Pichia*, suggesting these yeasts are key fungal genera associated with flavor variation in fermented camel milk. *Dekkera* and *Cyberlindnera* exhibited no clear correlation with the differential flavor compounds.

## 4. Discussion

This investigation carried out a systematic comparison of the microbial diversity present in naturally fermented camel milk obtained from three significant production areas within Xinjiang: Dabancheng, Hami, and Keping. The findings revealed that, although every specimen contained core bacterial phyla, namely *Firmicutes* and *Proteobacteria*, genus-level variation and a significance level of adjusted *p* < 0.05 were observed.

Earlier studies have documented substantial fluctuations related to season and geography in the bacterial composition and variety of camel milk throughout Xinjiang [12]. This observation is consistent with a fundamental principle in fermented dairy microbiology, which holds that both geographic provenance and manufacturing techniques jointly influence the microbiota of naturally soured milk [13]. An examination involving 142 raw milk specimens collected from seven different animal species in Xinjiang further corroborated the existence of source-dependent differences in milk microbial populations [14]. Likewise, research conducted on naturally fermented camel milk originating from the Alxa region of Inner Mongolia reported distinct bacterial and fungal diversity, with predictive analyses of gene functions uncovering its metabolic capabilities [2]. A more recent investigation (2025) went on to isolate and characterize lactic acid bacteria capable of metabolizing oligosaccharides from Xinjiang fermented camel milk, while also assessing diversity variations among samples from Dabancheng, Hami, and Keping [15]. These location-based disparities are probably attributable to two main factors.

To begin with, selective pressures exerted by the environment are critically important. Investigations into a range of conventional fermented foods consistently demonstrate that ecological factors drive the divergence of microbial community structures [16]. Within fermentation systems, non-living conditions, including temperature, acidity (pH), and water availability, interplay with biological interactions among microorganisms to shape the progression of community succession [17,18]. In the context of camel milk, the stage of lactation exerts a notable influence on probiotic levels, with milk produced during early lactation containing a greater abundance of microbes compared to that from later stages [19].

Dabancheng, situated in the cooler and more humid outskirts of Urumqi, produced specimens that were enriched with psychrotrophic or facultatively anaerobic bacteria, such as *Rahnella aquatilis*, alongside elevated populations of *Dekkera*. We hypothesize that the observed regional differences in microbial composition may be influenced by climatic factors (e.g., temperature, humidity) and environmental selection pressures. However, these factors were not directly measured in the present study, and future investigations incorporating environmental metadata are needed to test these hypotheses. For example, the higher abundance of psychrotrophic taxa in Hami samples could potentially be related to lower ambient temperatures in this region, but this remains speculative without direct temperature measurements. This latter organism is frequently linked to contamination by wild yeasts during open-air fermentation and has the capacity to generate phenolic flavor compounds, including 4-ethylguaiacol. By contrast, the dry and intensely hot conditions characteristic of Hami led to specimens containing high levels of *Pseudomonas* and *Pseudomonas fragi*. These bacterial species are commonly found in soil and water sources and were probably introduced through milking equipment or surrounding environmental contamination [20].

As a region with a longstanding nomadic tradition, Keping depends more heavily on naturally occurring environmental microbes for inoculation. This practice has resulted in the predominance of *Lactococcus* and *Serratia*, a phenomenon that mirrors the co-evolutionary interplay among the environment, the microbiome, and the host.

Secondly, disparities in fermentation methodologies also contribute to the observed variation. In Dabancheng and Hami, production is predominantly carried out using household workshop-style techniques, employing wooden or ceramic vessels. These container materials encourage the adherence and multiplication of microorganisms across successive batches. By contrast, the Keping area frequently preserves the age-old custom known as “backslopping,” whereby a portion of a previously fermented batch is used to initiate fermentation in fresh milk. This non-uniform inoculation approach probably results in the progressive enrichment of particular strains, such as *Klebsiella oxytoca*.

The PLSR model helped clarify the contribution of key microbial genera to flavor substances, revealing part of the microbiological basis for flavor development. As a core functional genus across all samples, the abundance of *Lactobacillus* showed a notably positive association with 2,3-butanedione, a compound known for its buttery aroma (*p* < 0.01). Notably, Lactobacillus abundance showed a significant positive correlation with 2,3-butanedione concentration (Pearson r = 0.86, *p* = 0.004), as further supported by the PLSR model (VIP = 1.34, Factor 1 loading = 0.72), indicating its potential role in diacetyl formation during fermentation. This finding aligns with the recognized metabolic characteristics of Lactobacillus, which generates pyruvate through glycolysis, a precursor that can subsequently be transformed into diacetyl [21]. Variations in functional genes among strains such as those found in *Lactobacillus delbrueckii* subsp. *Bulgaricus* directly influence their capacity to produce flavor compounds. Furthermore, multilocus sequence typing has uncovered links between particular genetic profiles and both fermentation duration and flavor characteristics [22]. Strains belonging to the *Lactobacillus* caseigroup demonstrated distinct flavor-generating abilities in experimental model systems; differences in their efficiency of amino acid metabolism led to substantial variations in the production yields of aldehydes, alcohols, and esters [23].

Of particular interest, the identification of *Gluconacetobacter rhaeticus* in Dabancheng specimens was associated with distinctive flavor substances, including 3-decyn-2-ol. This observation implies that acetic acid bacteria might take part in ester formation through the oxidation of ethanol into acetaldehyde and acetic acid. *Pseudomonas* reached its greatest abundance in Hami samples, yet it exhibited an inverse relationship with the majority of esters. This phenomenon could be attributed to the lipases and proteases secreted by *Pseudomonas*, which are capable of accelerating fatty acid oxidation and generating undesirable flavor compounds such as hexanal [24]. Research has confirmed that *Pseudomonas* strains, especially *P. fluorescens* and *P. fragi*, isolated from raw milk, produce heat-resistant lipases and proteases that retain their activity even after pasteurization, persistently breaking down milk fat into free fatty acids. These liberated fatty acids can then undergo β-oxidation, yielding short-chain aldehydes that impart oxidized and rancid sensory notes [25]. The negative correlation between Pseudomonas and esters could result from competitive exclusion of ester-producing organisms.

Yeasts utilize branched-chain amino acids as sources of nitrogen, transforming them through transaminase activity into α-keto acids. These intermediates are subsequently decarboxylated to form aldehydes, which are then reduced to higher alcohols. Such alcohols can undergo esterification to generate fruity esters. Studies conducted on fermented yak milk have substantiated the substantial role played by yeasts in the production of both alcohols and esters [5]. The observed positive associations between *Kazachstania*/*Candida* and alcohols/esters lend further support to this metabolic route. Acetic acid bacteria, exemplified by *Gluconacetobacter rhaeticus*, convert ethanol into acetaldehyde and acetic acid via membrane-associated dehydrogenase enzymes. Investigations involving synthetic microbial communities in Baijiu fermentation have corroborated analogous metabolic cross-feeding interactions, wherein intermediate compounds generated by one microorganism serve as substrates for the synthesis of flavor substances by another [26]. Such mutually beneficial relationships founded on the exchange of metabolites are prevalent in natural fermentation environments and can boost the output of particular flavor compounds [27]. These findings align with the classical Ehrlich pathway, which describes the conversion of amino acids into higher alcohols and esters within fermented dairy systems [28].

Nevertheless, the elevated prevalence of *Dekkera anomala* in Dabancheng specimens did not demonstrate any meaningful association with flavor compounds. One possible explanation is that the metabolic activity of *Dekkera* predominantly takes place during later stages of fermentation, which may not have been adequately represented by our chosen single sampling point [29]. *Dekkera* is known for its typically sluggish growth, and its metabolic contributions tend to be concentrated in the later phases of fermentation or during aging. In the context of sake brewing, chromosomal aneuploidy has been shown to influence the timing of its metabolic processes [30]. Investigations into intraspecies microdiversity suggest that a single microbial species can manifest divergent metabolic characteristics depending on the ecological conditions it encounters [31]. Consequently, the metabolic influence of *D. anomalain* Dabancheng specimens might necessitate a prolonged fermentation period to become fully apparent.

The ROAV assessment revealed that 2,3-butanedione served as the most prominent flavor contributor, achieving an ROAV exceeding 100 in Dabancheng and Keping samples, while in Hami samples the ROAV was 35.68 (Table 2). The ROAV assessment revealed that 2,3-butanedione served as the most prominent flavor contributor, achieving an ROAV exceeding 100 across all fermented camel milk specimens. Beyond 2,3-butanedione, alcohols such as 1-octen-3-ol (mushroom-like, earthy) and 3-methyl-1-butanol (malty, pungent) contributed to the complex aroma matrix, with the former being particularly abundant in Hami samples. Esters, especially ethyl heptanoate (fruity, pineapple) and ethyl nonanoate (floral, rose), were key to the fruity character of Dabancheng samples, consistent with the higher ester relative abundance observed in that region. These findings collectively illustrate that the volatile flavor profile of fermented camel milk is a composite of multiple compound classes, each imparting distinct sensory notes. This finding aligns with the elevated lactose and whey protein levels characteristic of camel milk, which furnish the necessary substrates for diacetyl biosynthesis by lactic acid bacteria [32]. Research indicates that moderate (mesophilic) fermentation conditions promote greater diacetyl accumulation compared to high-temperature fermentation, owing to the reduced activity of diacetyl reductase at lower temperatures [33]. The relatively abundant lactose content present in camel milk provides an ample glycolytic substrate, thereby facilitating pyruvate generation and consequently supplying a greater quantity of precursor for the branching reaction catalyzed by α-acetolactate synthase [34].

Consistent with Section 3, Hami samples generally exhibited the lowest bacterial and fungal alpha-diversity values among the three regions. This observation may be linked to the higher microbial diversity in these samples, with a Shannon index reaching 3.82. Such enhanced diversity can foster complex community interactions, leading to more elaborate metabolic networks [35]. The dry and intensely warm climate of Hami likely facilitates the introduction of *Pseudomonas* through milking apparatus and water sources, thereby promoting a more vigorous lipolysis–oxidation pathway. By contrast, the absence of certain aldehydes and ketones in Keping specimens could be attributed to the lower ambient fermentation temperatures typical of that area, averaging 8 to 12 °C annually, which may suppress secondary microbial metabolism [36]. When yeasts utilize amino acids as nitrogen sources, they inadvertently generate flavor-active substances through the Ehrlich pathway, converting amino acids into volatile alcohols and esters that contribute fruity and floral sensory notes.

Investigations into traditionally fermented yak milk from Ganan have directly validated the link between microbial community structure and flavor compounds. In those studies, fungal populations, particularly yeasts, demonstrated notably positive associations with alcohols and esters [5]. Dynamic analyses of kefir fermentation have additionally corroborated the synchronized relationship between yeast population growth and rising concentrations of alcohols and esters over time [37]. A reinforcing loop exists between microbial diversity and the intricacy of metabolic networks: greater diversity, as observed in Hami specimens, creates a larger number of cross-feeding junctions. This arrangement enables metabolites generated by one bacterial species to serve as substrates for another, facilitating the synthesis of increasingly complex compounds [38]. Such a cascade-like metabolic network permits communities with high diversity to generate a broader spectrum of flavor substances. Although carboxylic acids were detected at low levels across all regions, their limited abundance may reflect rapid conversion to esters via alcohol acyltransferase activity or utilization in other metabolic pathways [39].

For the very first time, this investigation approached naturally fermented camel milk collected from various Xinjiang regions as a unified research subject. Through integrated microbiome and volatile profiling correlation analysis, it unveiled the intricate interaction network connecting microbial communities, metabolites, and flavor. In contrast to earlier studies that concentrated on individual geographic areas [40], this regional comparative viewpoint more accurately captures the ecological complexity inherent in traditional fermented dairy products. Additionally, potentially functional microorganisms identified through the PLSR model, *Gluconacetobacter rhaeticus* and *Kazachstania* pseudohumilis, emerged as preliminary candidate taxa based on statistical associations. Their suitability as starter cultures requires isolation, characterization, and experimental validation of their functional roles in flavor generation [41]. Before the proposed microorganisms can be considered suitable for industrial application as direct vat set starter cultures, extensive validation is required [42]. Encapsulation technologies may further enhance strain viability and stability during processing and storage.

Factors such as camel breed, diet, and lactation stage also influence raw milk composition, thereby indirectly affecting microbial metabolic activity and flavor development. Research has documented substantial variations in both microbial populations and volatile organic compounds present in camel milk at different stages of lactation [43]. The fundamental composition of camel milk is characterized by low fat content, high levels of unsaturated fatty acids, and the absence of β-lactoglobulin, which sets it apart from bovine milk and restricts the range of metabolic substrates accessible to microorganisms [44]. Studies have confirmed that synthetic microbial communities can enhance fermentation dynamics and flavor attributes compared with inoculations relying on single strains [45]. Within the fermented camel milk system, a minimal functional community could be engineered based on strains showing positive correlations in the PLSR analysis, for instance, *Lactobacillus* and *Gluconacetobacter*, to progressively assess the flavor contribution of each constituent member. The essence of designing microbial communities lies in constructing functional consortia by combining metagenomic mapping with an appreciation of social behaviors such as quorum sensing.

From a critical standpoint, while our multi-omics approach successfully identified statistically significant correlations between specific microbial taxa and volatile flavor compounds, we acknowledge that correlation does not imply causation. The PLSR model, though robust, cannot fully account for the complex web of metabolic interactions, including cross-feeding, competition, and inhibition that occur in situ. For instance, the positive association between Lactobacillus and 2,3-butanedione may reflect not only direct diacetyl production by Lactobacillus but also indirect effects mediated by pH reduction or substrate availability altered by other community members. Similarly, the negative correlation between Pseudomonas and esters could result from competitive exclusion of ester-producing organisms rather than direct enzymatic degradation. Furthermore, our reliance on DNA-based amplicon sequencing provides compositional data but does not capture the active metabolic state of the microbiota. Metatranscriptomic or metaproteomic analyses would be necessary to confirm which taxa are actively expressing flavor-related genes. Another limitation is the small sample size (*n* = 3 per region), which, while sufficient for exploratory analysis, limits the generalizability of our findings. Finally, the absence of carboxylic acids and heterocyclic compounds in all samples warrants caution—this may partly reflect the limitations of the SPME-GC×GC-TOF MS method, which favors semi-volatile compounds, rather than a true absence of these classes. Future studies should integrate complementary extraction techniques (e.g., solvent-assisted flavor evaporation) and sensory analysis (e.g., GC–olfactometry) to validate and extend our findings. Despite these caveats, our study provides a valuable roadmap for hypothesis-driven strain selection and synthetic community design [46].

This study has several limitations. First, the key microbial strains were not isolated, purified, or validated through fermentation trials; therefore, the observed correlations between microorganisms and flavor require confirmation via pure-culture experiments. Secondly, non-microbial elements, including camel breed and feed composition, were not assessed for their potential effects on flavor. Thirdly, the volatile substances detected through GC-MS represent only a subset of the complete flavor profile; forthcoming investigations should incorporate GC–olfactometry and sensory genomics to achieve a more refined flavor analysis. Future research endeavors ought to concentrate on: (1) isolating and characterizing the functional strains identified by the PLSR model, along with evaluating their aroma-generating properties; (2) constructing synthetic microbial communities (SynComs) to replicate natural fermentation and verify the mechanisms underlying flavor formation; and (3) integrating metabolomics with sensory evaluation to develop a digital flavor assessment system tailored to fermented camel milk.

## 5. Conclusions

This investigation carried out a thorough examination of the microbial diversity and community organization present in naturally fermented camel milk obtained from the Dabancheng, Hami, and Keping areas of Xinjiang, as well as their associations with volatile flavor compounds. The outcomes revealed pronounced regional divergence in both bacterial and fungal populations. With regard to bacterial communities, although *Firmicutes* and *Proteobacteria* constituted the prevailing phyla across every specimen, the dominant genera and species differed considerably. Specimens from Dabancheng were chiefly distinguished by *Lactobacillus*, *Lactococcus*, and *Rahnella aquatilis*; those from Hami were predominantly characterized by *Lactobacillus*, *Lactococcus*, and *Pseudomonas fragi*; whereas samples from Keping consisted mainly of *Lactococcus*, *Serratia*, and *Klebsiella oxytoca*. Key microbial genera, including *Lactobacillus*, *Lactococcus*, *Acetobacter*, *Pseudomonas*, and Dekkera, were significantly correlated with specific volatile compounds. Notably, functionally specialized strains, such as the acetic acid-producing *Gluconacetobacter rhaeticus,* were identified exclusively in Dabancheng specimens, indicating a prospective involvement in molding the local flavor profile. Fungal communities likewise displayed marked location-specific variation. *Dekkera* *anomala* represented the predominant fungus in Dabancheng samples, with a relative abundance exceeding 54%, whereas the leading taxa in Hami and Keping belonged to the genera *Kazachstania* and *Cutaneotrichosporon*, respectively.

Examination of volatile flavors confirmed that disparities in microbial community architecture corresponded to distinct metabolic signatures. Dabancheng specimens were abundant in esters, alcohols, and ketones, while Hami and Keping samples were predominantly defined by hydrocarbons and alcohols. Through the application of relative odor activity value (ROAV) analysis, 11 key aroma compounds, each with an ROAV of 1 or greater, were identified. 2,3-Butanedione, which contributes a buttery sensory note, emerged as the most impactful flavor contributor across all specimens. Hami samples contained the largest number of key aroma compounds—nine in total—substantially exceeding the four found in both Dabancheng and Keping specimens. This observation may be associated with the more intricate microbial community structure present in Hami. Partial least squares regression (PLSR) modeling successfully established quantitative relationships between particular microbial taxa and essential flavor substances. Among the bacteria, *Lactobacillus*, *Rahnella*, and *Gluconacetobacter* exhibited notably positive associations with compounds such as propene and 3-decyn-2-ol, pointing to their probable direct involvement in generating these flavors. Conversely, *Pseudomonas*, *Lactococcus*, and *Klebsiella* displayed inverse correlations with several esters, hinting at possible metabolic rivalry or substrate utilization. Within the fungal domain, *Kluyveromyces*, *Candida*, and *Kazachstania* were significantly linked to various characteristic flavor compounds, whereas the highly abundant *Dekkera* showed no meaningful correlation. This suggests that the fungal contribution to flavor formation may depend more on specific metabolic activities than on sheer numerical abundance. Their statistical association with these flavors suggests potential contributions that warrant experimental validation through pure-culture fermentation studies.

In conclusion, this study systematically delineated the connections among geographic origin and microbial community structure. This integrated microbiome and volatile profiling analysis reveals region-specific microbial communities and flavor profiles. The results indicate that regional environmental influences and conventional fermentation techniques collaboratively mold unique microbial consortia that drive the creation of location-specific volatile flavor patterns. This work offers novel perspectives on the interactions among microbiota, metabolites, and flavor within traditional fermented dairy products. Crucially, these microorganisms represent potential key functional taxa whose roles in flavor generation require experimental confirmation. Future work should focus on constructing synthetic microbial communities (SynComs) based on these positive correlations to validate their causal roles in flavor formation and ultimately design robust, commercially viable starter cultures that can replicate the unique flavor profiles of each region. This advances the efforts toward standardization, quality assurance, and large-scale industrial production of fermented camel milk.

## Figures and Tables

**Table 1:** Sample information sheet.

Sample Number	Sample	Sampling Position
A-1	Fermented camel milk	Dabancheng (A)
A-2	Fermented camel milk	Dabancheng (A)
A-3	Fermented camel milk	Dabancheng (A)
B-1	Fermented camel milk	Hami (B)
B-2	Fermented camel milk	Hami (B)
B-3	Fermented camel milk	Hami (B)
C-1	Fermented camel milk	Keping (C)
C-2	Fermented camel milk	Keping (C)
C-3	Fermented camel milk	Keping (C)

**Table 2:** Relative content statistics of high ROAV compounds.

Name	A_ROAV	B_ROAV	C_ROAV
1-Octen-3-ol	0.00140294	0.003621516	0.003278212
1-Butanol, 3-methyl-	0.158631311	0.38175371	0.825631341
Isopropyl alcohol	7.93997 × 10^−8^	3.40353 × 10^−5^	2.17259 × 10^−6^
1-Propanol, 2-methyl-	0.013105684	0.011078862	0.008626419
Ethanol	9.78089 × 10^−5^	6.34102 × 10^−5^	8.98825 × 10^−5^
1-Propanol	2.8285 × 10^−7^	4.51146 × 10^−7^	5.6569 × 10^−8^
Benzyl alcohol	4.00951 × 10^−7^	1.96535 × 10^−8^	1.82208 × 10^−8^
1-Hexanol, 2-ethyl-	0.033052019	0.028331093	0.040417562
2-Nonanol	0.000138498	0.001806343	1.60195 × 10^−5^
2-Heptanol	8.05223 × 10^−5^	0.000166103	1.3484 × 10^−5^
2-Butanol	0.000159509	0.001375964	0.00015205
1-Octanol	0.005009852	0.023434331	0.016991337
1-Pentanol	0.001304355	0.003872219	0.00570503
1-Hexanol	0.01747427	0.066453856	0.025554319
1-Butanol	0.000577357	0.00056746	0.000332223
2-Nonenal, (E)-	4.456636406	100	2.346033944
2-Octenal, (E)-	0.129122306	7.539556587	0.180249792
Octanal	3.17599 × 10^−5^	0.00975381	0.000903624
Hexanal	0.0001314	0.005253028	0.000391037
Heptanal	0.851986513	21.90465262	0.806922413
Benzeneacetaldehyde	0.008425526	0.024201871	0.026173665
Butanal, 3-methyl-	0.000231586	0.000723687	0.000300197
Naphthalene	0.003073977	0.000769254	0.000215777
Benzaldehyde	0.168216048	0.06079117	0.000970117
Toluene	0.00040433	0.000594148	0.000512108
p-Cresol	1.32333 × 10^−6^	5.00209 × 10^−5^	1.68685 × 10^−5^
Phenol	1.76444 × 10^−5^	0.000856033	0.000284999
Benzene	2.12725 × 10^−5^	1.3704 × 10^−5^	1.02454 × 10^−5^
Benzene, 1,4-dichloro-	8.93329 × 10^−5^	3.1967 × 10^−5^	4.52343 × 10^−5^
Benzene, 1,2,4-trimethyl-	0.000368025	0.0002232	5.19417 × 10^−5^
Acetic acid	0.091295381	2.718382556	0.608404797
Butanoic acid, 3-methyl-, ethyl ester	0.000151393	0.000188802	5.45699 × 10^−5^
Propanoic acid, ethyl ester	3.33015 × 10^−7^	5.13066 × 10^−6^	3.38258 × 10^−7^
Isobutyl acetate	0.000673859	0.008636979	0.000352749
Ethyl acetate	0.00980125	0.005180742	0.003547103
Propanoic acid, 2-methyl-, ethyl ester	0.00393212	0.001067499	1.03047 × 10^−5^
1-Butanol, 3-methyl-, acetate	0.000398835	0.000682318	0.000393237
n-propyl acetate	0.000180539	0.002251167	9.56922 × 10^−5^
Tetradecanoic acid, ethyl ester	0.000453257	7.93779 × 10^−6^	1.02168 × 10^−6^
Dodecanoic acid, ethyl ester	0.001036113	6.2555 × 10^−6^	1.25207 × 10^−6^
Propanoic acid, 2-hydroxy-, ethyl ester	6.2031 × 10^−10^	1.97028 × 10^−6^	3.62196 × 10^−7^
Nonanoic acid, ethyl ester	1.54575 × 10^−5^	2.61508 × 10^−7^	1.47181 × 10^−8^
Dibutyl phthalate	0.000126017	6.0029 × 10^−5^	4.29454 × 10^−5^
Hexanoic acid, ethyl ester	0.006810184	0.000969091	0.000274607
Butanoic acid, 2-methyl-, ethyl ester	0.011757462	0.00067247	0.000528229
Butanedioic acid, diethyl ester	2.24928 × 10^−10^	6.43148 × 10^−10^	5.75445 × 10^−10^
Decanoic acid, ethyl ester	0.001460985	2.40912 × 10^−5^	1.96391 × 10^−6^
Heptanoic acid, ethyl ester	2.82173 × 10^−6^	9.28505 × 10^−8^	3.52547 × 10^−9^
Butanoic acid, ethyl ester	0.007250877	0.0005103	0.000299365
Pentanoic acid, 2-hydroxy-4-methyl-, ethyl ester	0.000523914	0.000571072	3.30998 × 10^−6^
1-Propanol, 3-(methylthio)-	9.25074 × 10^−6^	8.52082 × 10^−6^	2.24203 × 10^−6^
Propene	4.05634 × 10^−7^	3.12948 × 10^−7^	1.79228 × 10^−8^
Nonane	3.45216 × 10^−8^	3.95685 × 10^−6^	3.60941 × 10^−6^
Pentane, 2-methyl-	1.86384 × 10^−7^	5.26064 × 10^−6^	5.9341 × 10^−6^
1-Octen-3-one	0.48232437	4.136669865	3.355863587
Acetophenone	0.023093698	0.000250634	0.010612023
3-Heptanone, 5-methyl-	1.88514 × 10^−6^	2.0111 × 10^−7^	1.43032 × 10^−6^
2-Pentanone	0.000348272	1.63079 × 10^−6^	0.000559773
2-Undecanone	0.750795063	1.304751667	0.010647773
2-Nonanone	0.00064766	0.003085337	0.0004061
2-Heptanone	0.027271835	0.180404519	0.041612545
2,3-Butanedione	100	35.68047633	100
a-Pinene	7.274180149	2.18608079	0.000772851
Myrcene	2.55842 × 10^−6^	7.2609 × 10^−6^	1.16846 × 10^−6^
n-Decanoic acid	1.19418 × 10^−5^	2.30857 × 10^−6^	5.09833 × 10^−7^
Linalool	3.08266 × 10^−5^	3.84267 × 10^−5^	1.8302 × 10^−5^
p-Cymene	0.014134676	0.000100883	1.25358 × 10^−5^
Nonanoic acid	2.88055 × 10^−6^	1.52215 × 10^−6^	1.13142 × 10^−6^
Trichloromethane	0.000165189	7.95576 × 10^−5^	7.57975 × 10^−5^
Benzothiazole	5.76953 × 10^−7^	8.35418 × 10^−8^	1.649 × 10^−7^
2H-Pyran-2-one, tetrahydro-6-pentyl-	6.10004 × 10^−6^	1.3268 × 10^−5^	1.90811 × 10^−6^
2H-Pyran-2-one, tetrahydro-6-propyl-	1.27956 × 10^−6^	4.87167 × 10^−7^	1.15928 × 10^−7^
2(3H)-Furanone, 5-hexyldihydro-	0.003605543	0.00450455	0.000138725
Butyrolactone	6.45013 × 10^−5^	0.001432956	2.90995 × 10^−5^
Furan, 2-ethyl-	0.00019953	0.00942286	0.002946324
Furan, 2-pentyl-	6.738426792	21.22775834	10.40061579
Octanoic acid, ethyl ester	0.005546061	0.00062741	3.93572 × 10^−5^
Dimethyl trisulfide	0.007182436	0.004588045	0.005260959
Disulfide, dimethyl	0.068915318	0.010584846	0.007386081
Boron trifluoride	3.41478 × 10^−6^	4.578 × 10^−7^	2.22999 × 10^−6^
Phenylethyl alcohol	7.68609 × 10^−5^	3.80513 × 10^−5^	0.000253112

## Data Availability

The original contributions presented in this study are included in the article. Further inquiries can be directed to the corresponding author.

## Abbreviations

The following abbreviations are used in this manuscript:

GC×GC-TOF MS	Comprehensive Two-Dimensional Gas Chromatography–Time-of-Flight Mass Spectrometry
VFCs	Volatile Flavor Compounds
OTU	Operational Taxonomic Unit
ROAV	Relative Odor Activity Value
PCA	Principal Component Analysis
PCoA	Principal Coordinates Analysis
PLSR	Partial Least Squares Regression
HCA	Hierarchical Cluster Analysis
VIP	Variable Importance in Projection
FC	Fold Change
SPME	Solid-Phase Microextraction
DVB/CAR/PDMS	Divinylbenzene/Carboxen/Polydimethylsiloxane
EI	Electron Ionization
DVS	Direct Vat Set
SynComs	Synthetic Microbial Communities
